# Short- to Long-Term Effects of Virtual Reality on Motor Skill Learning in Children With Cerebral Palsy: Systematic Review and Meta-Analysis

**DOI:** 10.2196/42067

**Published:** 2023-09-12

**Authors:** Seyma Kilcioglu, Benoît Schiltz, Rodrigo Araneda, Yannick Bleyenheuft

**Affiliations:** 1 Institute of Neuroscience Université Catholique de Louvain (UCLouvain) Brussels Belgium; 2 Exercise and Rehabilitation Science Institute, School of Physical Therapy, Faculty of Rehabilitation Science Universidad Andrés Bello Santiago Chile

**Keywords:** cerebral palsy, virtual reality, motor skill learning, long-term effect, daily life activities, motor functions

## Abstract

**Background:**

Many studies have started integrating virtual reality (VR) into neurorehabilitation for children with cerebral palsy (CP). The results of the effects of VR on motor skill learning, including the short- to long-term results of relevant studies, must be pooled in a generic framework.

**Objective:**

This systematic review and meta-analysis aimed to investigate the short- to long-term effects of therapies including VR on motor skill learning in children with CP.

**Methods:**

Two examiners followed the inclusion and exclusion criteria of the “Participant, Intervention, Control, and Outcome” framework. Randomized controlled trials (RCTs) and non-RCTs were considered if they compared VR-included interventions with control groups on motor functions and daily life activities in children with CP. PubMed, ScienceDirect, Embase, and IEEE Xplore databases were searched. The modified Downs and Black assessment was used to assess the methodological quality of the included studies. Meta-analyses and subgroup analyses for RCTs were conducted whenever possible.

**Results:**

A total of 7 RCTs, 2 non-RCTs, and 258 children with CP were included. The priority focus of 78% (7/9) of the studies was upper limb functions. There was a significant short-term effect of adding VR to conventional therapies on upper limb functions when compared with conventional therapies (*P*=.04; standardized mean difference [SMD]=0.39, 95% CI 0.01-0.76). The overall medium- to long-term effects showed a trend toward favoring the VR group, although the difference was not statistically significant (*P*=.06; SMD=0.37, 95% CI −0.02 to 0.77). For balance (*P*=.06; SMD=1.04, 95% CI −0.04 to 2.12), gross motor functions (*P*=.30; SMD=2.85, 95% CI −2.57 to 8.28), and daily life activities outcomes (*P*=.21; SMD=0.29, 95% CI −0.16 to 0.74), the overall effect in the short term also showed a trend toward favoring the VR group, but these results were not statistically significant.

**Conclusions:**

VR seems to have additional benefits for motor skill learning in children with CP. Studies with follow-up outcomes of VR training focusing on balance and gross motor functions in patients with CP were quite limited. Future research on balance and gross motor function outcomes should target particularly long-term results of therapies including VR on motor skill learning.

**Trial Registration:**

PROSPERO International Prospective Register of Systematic Reviews CRD42021227734; https://www.crd.york.ac.uk/prospero/display_record.php?ID=CRD42021227734

## Introduction

### Background

Cerebral palsy (CP), the leading motor disability in childhood with a worldwide prevalence of 2 per 1000 live births, is a group of permanent movement and posture disorders caused by an early brain lesion during pregnancy, childbirth, or shortly after birth [1,2]. In addition to the motor consequences, sensory and cognitive functions affecting daily life activities may also be impaired [2]. From the rehabilitation perspective, motor skill learning–based approaches have shown encouraging efficacy in improving motor functions in children with CP in recent years [3]. Interventions based on motor skill learning principles (more intensity, performance of daily living activities, repeating functional tasks, individually tailored, progressively challenging, and motivating feedback) have been shown to promote persistent motor skill acquisition and neuroplasticity (practice-induced brain changes) [4,5]. In addition, it is now well established that sustaining children’s attention, interest, and enjoyment for a long time is one of the critical factors, but it is also challenging for optimizing rehabilitation outcomes and neuroplastic changes [6]. Consequently, researchers have focused on how to foster neuroplastic changes and functional improvements with more intensity and diversity of practice without a trade-off in terms of entertainment.

Thus, with the results of recent studies and the increase in access to computer-assisted technologies, the use of virtual reality (VR) as a means of neurorehabilitation for children with CP has been encouraged. VR refers to a computer-generated simulation that may vary in complexity and reality owing to the rapidly evolving nature of technology [7,8]. The VR environment can be experienced through a variety of display hardware, including standard monitors, flat screens, projection screens, and head-mounted displays. The method of interacting with the VR system may also vary from simple activation of computer keyboard keys, mouse, or joystick to more advanced motion camera interfaces [9,10]. Consequently, VR devices used in rehabilitation can be classified as immersive, semi-immersive, and nonimmersive depending on the level of immersion [11,12]. Furthermore, certain devices are specifically designed for rehabilitation purposes, whereas commercially available devices and games are also used in the rehabilitation of various disorders. A systematic review on the clinical utility of VR in neurorehabilitation reported that CP is the second most studied neurological disorder in VR rehabilitation [13]. In addition, You et al [14] and Golomb et al [15] showed cortical reorganization after VR therapy in children with CP and the association of the cortical changes with functional abilities. The advantage of using VR in rehabilitation is not only to increase entertainment with playful interactive games but also to have games with individually tailored, progressively adjustable difficulty levels and to provide active repetition in an enriched environment [9]. Thus, with these advantages, VR may well correspond to the challenges in rehabilitation such as the use of motor skill learning principles to develop long-lasting motor skill acquisition in children.

### Objectives

Several systematic reviews investigated the effectiveness of VR on upper and lower limb functions, gross motor functions, and postural control in children with CP in the short term, that is, immediately after treatment [16-18]. They showed a promising potential of adding VR into CP interventions for improving motor functions. However, the long-term effects of VR interventions on motor skill learning remain unclear, although durable retention of acquired skills is one of the crucial factors for rehabilitation objectives. Consequently, this systematic review and meta-analysis was conducted to examine the effect of VR on motor functions and daily life activities of children with CP from the short term (immediately after the intervention) to the medium term (≥1 week to <12 weeks after the end of the intervention) or long term (≥12 weeks after the end of the intervention).

## Methods

### Protocol and Registration

We followed the guidelines of the PRISMA (Preferred Reporting Items for Systematic Reviews and Meta-Analyses) 2020 statement to conduct this systematic review and meta-analysis and reported our results according to PRISMA 2020 checklist [19]. The protocol of this review was registered in the PROSPERO database (CRD42021227734).

### Eligibility Criteria

Inclusion criteria of the studies were defined after discussion among all authors according to the PICO (Population, Intervention, Comparison, and Outcome) framework [20]. As a result, studies (1) with a population of children with CP aged <18 years; (2) using VR for main intervention; (3) comparing the experimental group (therapy involving VR) with any control group (another intervention except VR, nonintervention, placebo effect, or different cohorts); and (4) including baseline, postintervention, and follow-up assessments by using outcome measures on motor functions and daily life activities were included. We excluded studies if they (1) involved populations other than children with CP aged <18 years; (2) used VR only for assessment or focused on the development of VR devices; (3) used different outcome measures than on motor functions and daily life activities; (4) were not published in English; (5) were reviews, meta-analyses, commentaries, protocols, and conference abstracts; and (6) did not have follow-up assessment (ie, had only baseline and postintervention assessments). In this review, we decided to include studies involving commercially available VR devices or those specifically developed for therapy, regardless of the degree of immersive experience, type of display hardware, or the method of interaction with the VR system.

### Information Sources and Search Strategy

Two authors systematically and independently searched PubMed, ScienceDirect, Embase, and IEEE Xplore databases in September 2021 using predetermined keyword combinations, which were “virtual reality,” “virtual environment,” “videogaming,” “computer game,” “Kinect,” “Wii,” “PlayStation,” “cerebral palsy,” “CP,” “rehabilitation,” “therapy” and “motor skill learning.” The results of the search in the 4 databases were transferred to the EndNote (version 9; Clarivate), and duplicates were removed by 1 author. The search strategy used for each database is detailed in Multimedia Appendix 1. We did not apply any restriction regarding the date of publication for our search strategy.

### Study Selection Process

After detecting and eliminating duplicated studies obtained from the searches, 2 authors first screened titles and abstracts according to the inclusion and exclusion criteria. After several discussions and resolving the conflicts between authors, full texts of studies that have the potential to be included were obtained. Then, the eligibility of the studies was assessed using full-text screening. All screening and study selection processes were performed by the same 2 authors who were blinded to each other using Rayyan web-based software (Qatar Computing Research Institute) [21].

### Data Collection Process and Data Items

The following information was extracted by 2 authors independently to a predesigned Microsoft Excel (Microsoft Corp) sheet: characteristics of the study (first author, year of publication, and study design), characteristics of the participants (number of participants, CP type, age, Manual Ability Classification System [MACS], and Gross Motor Function Classification System [GMFCS] level), characteristics of the VR interventions and comparison interventions (name, duration, frequency, length, total hour, priority focus, and motor skill learning principles involved in experimental interventions), characteristics of outcomes measures (timing and measures), and main outcomes. In addition, to collect the data from the included studies, another predesigned Microsoft Excel sheet was used, with the following content: mean and SD of experimental (VR) and control groups at postintervention and follow-up points and sample sizes of both groups. When there was no information about the means and SDs of results, they were calculated from SE, CI, *t* test, or *P* value as recommended by the Cochrane handbook [22]. Disagreements between the data collectors were resolved by a discussion.

### Methodological Quality Assessment

Two authors independently assessed the methodological quality of the included studies using the modified Downs and Black checklist. This is a valid and reliable tool to assess the quality of both randomized controlled trials (RCTs) and non-RCTs [23]. Conflicts were resolved by discussion. This checklist comprises 27 items on the quality of reporting (10 items), external validity (3 items), internal validity (bias and confounding; 13 items), and statistical power (1 item).

The highest possible score for the original checklist was 32. However, as item 27 was complicated to score (from 0 to 5), a modified version of the scoring method was proposed, as observed in previous studies [17,24,25]. Thus, each item was scored as 0 (no) or 1 (yes), except for the fifth item that was scored as 0 (no), 1 (partially), or 2 (yes). Consequently, scores on the modified Downs and Black checklist corresponding to quality levels ranged between “excellent” (scores of 24-28), “good” (scores of 19-23), “fair” (scores of 14-18), and “poor” (scores of ≤13) [26,27].

### Effect Measures and Synthesis Methods

We conducted a meta-analysis for the RCTs that met the criteria for eligible design and outcomes. The results from the studies on motor functions and activities of daily living were extracted into a Microsoft Excel sheet. For continuous data, standardized mean difference (SMD) with a 95% CI, which refers to the effect size, was measured using a random effects model because of the different clinical tests used in the included studies. For the interpretation of effect sizes, we followed Cohen rule in which 0.2, 0.5, and 0.8 represent small, medium, and large effect sizes, respectively [28]. Statistical significance was set at .05, and statistical heterogeneity was examined using the *I*^2^ statistic. Heterogeneity was rated as low (*I*^2^≤25%), moderate (25%<*I*^2^<75%), or high (*I*^2^≥75%).

To explore whether the duration and intensity of VR training (more intensive: more therapy time per week, which is obtained by the higher frequency of sessions or longer duration of each therapy session) as well as the type of VR had some influence on the results, subgroup analyses were performed (when possible) according to the following criteria: duration (<30 min per session, ≥30 to <60 min per session, or ≥60 min per session), intensity (≤120 min per week or >120 min per week) and VR type (specifically developed for therapy or commercially available). The software Review Manager (version 5.4.1; Cochrane) for Windows was used to conduct the analyses of the included studies.

## Results

### Study Selection

**Figure 1:**
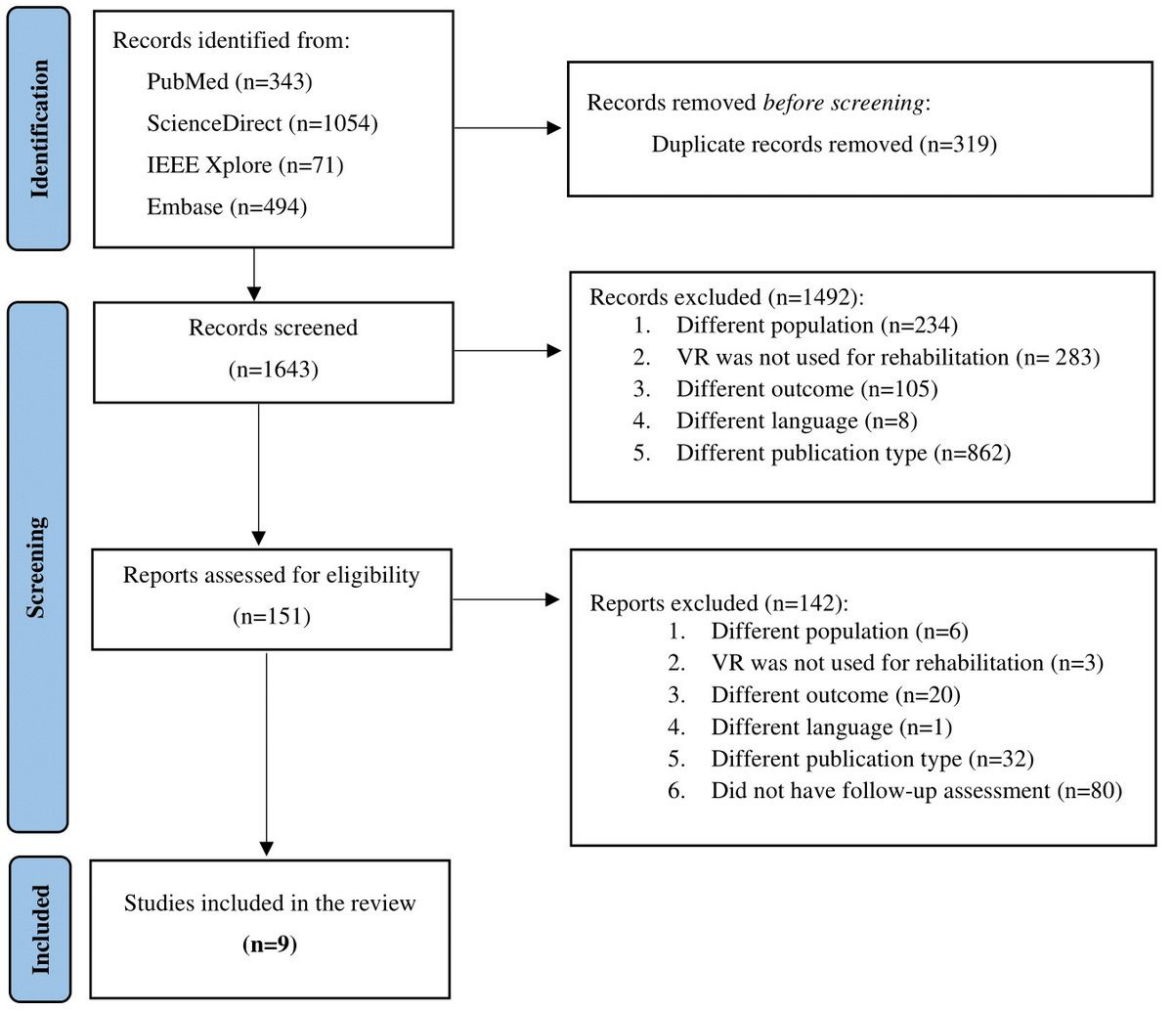
PRISMA (Preferred Reporting Items for Systematic Reviews and Meta-Analyses) flow diagram of the study selection process. VR: virtual reality.

Our database search resulted in 1643 studies after removing 319 duplicate records. After title and abstract screening, we excluded 1492 records for the reasons specified in Figure 1. Full texts of the remaining 151 studies were assessed for eligibility, and finally, 9 studies met the inclusion criteria, 7 (78%) of which were RCTs.

### Study Characteristics

#### Overview

The 9 included studies involved 258 children, 163 with unilateral CP and 95 with bilateral CP (BCP), aged between 3 and 18 years. Four studies reported the gross motor functions of children, classifying them from levels I to V according to the GMFCS [29-32]. Five studies classified children from level I to V regarding the use of their hands when handling objects in daily activities using the MACS [29,32-35]. The characteristics of the included studies are presented in Tables 1-3.

**Table 1:** List of study characteristics.

Study	Participants	Experimental group				Control group				Outcomes measures: timing and measures		Main outcomes
		Intervention	Duration, frequency, length, and total time of VR^a^	Priority focus	Intervention		Duration, frequency, length, and total time	Priority focus				
Choi et al [33], 2021	34 children with unilateral CP^b^ and 44 children with bilateral CP, age: 3-18 years	VR (RAPAEL Smart Kids) + conventional occupational therapy (stretching, strengthening, and task-oriented training)	30 min, 5 sessions, 4 weeks, and 10 h	Upper limb	Conventional occupational therapy		60 min, 5 sessions, 4 weeks, and 20 h	Upper limb	Baseline, postintervention, follow-up (8 +1 or −1 weeks after completion of intervention); MA-2^c^, ULPRS^d^, and PEDI-CAT^e^		More improvement in VR group in MA-2 and performance of activities of daily living domain of PEDI-CAT. Improvements were retained at follow-up.	
Preston et al [34], 2016	14 children with unilateral CP and 1 child with bilateral CP, age: 5-12 years	VR (computer-assisted arm rehabilitation gaming technology) + conventional therapy + botulinum toxin	7 min per session, 6 weeks, and 1 h 39 min	Upper limb	Conventional therapy + botulinum toxin		6 weeks	Upper limb	Baseline, postintervention, follow-up (6 weeks after completion of intervention); ABILHAND-Kids questionnaire, and COPM^f^		No improvement in both groups in ABILHAND-Kids, and no difference between groups at any time point. Improvement in all participants in COPM.	
Peper et al [35], 2013	6 children with unilateral CP, age: 7-12 years	VR (computer games with Lissajous feedback) + conventional therapy (if any)	30 min, 3 sessions, 6 weeks, and 9 h	Upper limb	Conventional therapy		6 weeks	—^g^	Baseline, postintervention, follow-up (6 weeks after completion of intervention); AHA^h^		No improvement at group level in AHA.	
Rostami et al [36], 2012	32 children with unilateral CP, age: 6-12 years	VR group: VR (E-Link Evaluation and Exercise System) + conventional therapy, VR + mCIMT^i^ group	90 min, 3 sessions, 4 weeks, and 18 h	Upper limb	Conventional therapy		30 min, 2 sessions, 4 weeks, and 4 h	Upper limb	Baseline, postintervention, follow-up (12 weeks after completion of intervention); PMAL^j^, and speed and dexterity subtest of BOTMP^k^		More improvement in VR + mCIMT group in PMAL and BOTMP. Improvements were retained at follow-up.	
Kassee et al [29], 2017	6 children with unilateral CP, age: 7-12 years	VR (Nintendo Wii)	40 min, 5 sessions, 6 weeks, and 20 h	Upper limb	Resistance training		36-48 min, 5 sessions, 6 weeks, and 18-24 h	Upper limb	Baseline, postintervention, follow-up (4 weeks after completion of intervention); MA-2, ABILHAND-Kids questionnaire, and grip strength		No statistical analysis to determine group differences.	
Psychouli and Kennedy [37], 2016	9 children with unilateral CP, age: 5-11 years	VR (computer game with a joystick) + mCIMT	20 min, 7 sessions, 4 weeks, and 9 h	Upper limb	mCIMT		4 weeks	Upper limb	Baseline, postintervention, follow-up (4 weeks after completion of intervention); MA, and QUEST^l^		More improvement in VR + mCIMT group in MA, QUEST. Improvement in MA was retained at follow-up.	
Decavele et al [30], 2020	32 children with bilateral CP, age: 6-15 years	VR (OpenFeasyo games with Wii Balance Board) + conventional therapy	At least 15-20 min, 2 sessions, 12 weeks, and approximately 5 h	Upper and lower limb, trunk control	Conventional therapy		32.6 min, 20.6 sessions (total),12 weeks, and approximately 11 h	—	Baseline, postintervention, after washout period (12 weeks after), after control period; GAS^m^, TCMS^n^, PBS^o^, and GMFM^p^-88		More improvement after intervention period (VR + conventional therapy) compared with control period (conventional therapy) in GAS, TCMS, PBS, GMFM-88. Improvements were not retained at follow-up.	
Pin and Butler, 2019 [31]	18 children with bilateral CP, age: 6-14 years	VR (TYROMOTION force plate) + conventional therapy	20 min, 4 sessions, 6 weeks, and 8 h	Postural control	Conventional therapy		6 weeks	—	Baseline, midpoint of intervention (week 3), postintervention (week 6), follow-up (6 weeks after completion of intervention); PRT^q^, GMFM-66, and 2-min walk test		No significant difference between the groups at any time and in any of the assessments. Improvements in both groups in GMFM-66 after 6 weeks of the intervention.	
Chiu et al [32], 2014	62 children with unilateral CP, age: 6-13 years	VR (Wii Sports Resort) + conventional therapy	40 min, 3 sessions, 6 weeks, and 12 h	Upper limb	Conventional therapy		6 weeks	—	Baseline, postintervention, follow-up (6 weeks after completion of the intervention); coordination (elbow and finger), grip strength, Nine-Hole Peg Test, JTTHF^r^, and FUS^s^		No significant difference between the groups in coordination, Nine-Hole Peg Test, and Jebsen-Taylor Test. Trend for the experimental group to have more grip strength and FUS quantity.	

^a^VR: virtual reality.

^b^CP: cerebral palsy.

^c^MA-2: Melbourne Assessment 2.

^d^ULPRS: Upper Limb Physician’s Rating Scale.

^e^PEDI-CAT: Pediatric Evaluation of Disability Inventory Computer Adaptive Test.

^f^COPM: Canadian Occupational Performance Measure.

^g^Not available.

^h^AHA: Assisting Hand Assessment.

^i^mCIMT: modified constraint-induced movement therapy.

^j^PMAL: Pediatric Motor Activity Log.

^k^BOTMP: Bruininks-Oseretsky Test of Motor Proficiency.

^l^QUEST: Quality of Upper Extremity Skills Test.

^m^GAS: Goal Attainment Scale.

^n^TCMS: Trunk Control Measurement Scale.

^o^PBS: Pediatric Balance Scale.

^p^GMFM: Gross Motor Function Measure.

^q^PRT: Pediatric Reach Test.

^r^JTHFT: Jebsen-Taylor Hand Function Test.

^s^FUS: Functional Use Survey.

**Table 2:** Motor skill learning principles of experimental interventions and results in upper limb functions and daily life activities.

Study	Experimental intervention	Motor skill learning principles involved in the experimental intervention	Changes in upper limb functions and daily life activities
Choi et al [33], 2021	Conventional occupational therapy + VR^a^ (specifically developed for therapy)	More intensity, performance of daily life activities, repeating specific functional tasks, individually tailored, progressively challenging training, and simultaneous feedback (auditory and visual)	^↑↑b^
Preston et al [34], 2016	Conventional therapy + botulinum toxin + VR (specifically developed for therapy)	—^c^	^→d^
Peper et al [35], 2013	Conventional therapy (if any) + VR (specifically developed for therapy)	Many repetitions of the movements, progressively challenging, and Lissajous feedback	^→^
Rostami et al [36], 2012	mCIMT^e^ + VR (specifically developed for therapy)	More intensity, performance of daily life activities, repetitive motor practice, individually tailored, progressively challenging training, and simultaneous feedback (auditory and visual)	^↑↑^
Kassee et al [29], 2017	VR (commercially available)	More intensity	No statistical analysis
Psychouli and Kennedy [37], 2016	mCIMT + VR (commercially available)	More intensity, performance of daily life activities, repeating specific functional tasks, individually tailored, and augmented motivating feedback	^↑↑^
Chiu et al [32], 2014	Conventional therapy + VR (commercially available)	Simultaneous feedback	^→^

^a^VR: virtual reality.

^b^More improvement (retained at follow-up) in the experimental group compared with the control group.

^c^Not available.

^d^No improvement in the experimental group or no significant difference between the groups (experimental and control groups).

^e^mCIMT: modified constraint-induced movement therapy.

**Table 3:** Motor skill learning principles of experimental interventions and results in balance, trunk control, gross motor functions, and daily life activities.

Study	Experimental intervention	Motor skill learning principles involved in the experimental intervention	Changes in balance, trunk control, and gross motor functions and daily life activities
Decavele et al [30], 2020	Conventional therapy + VR^a^ (specifically developed for therapy)	Individually tailored, and progressively challenging	^↑→^^b^
Pin and Butler [31], 2019	Conventional therapy + VR (specifically developed for therapy)	Progressively challenging	^→^^c^

^a^VR: virtual reality.

^b^More improvement (not retained at follow-up) in the experimental group compared with the control group.

^c^No improvement in the experimental group or no significant difference between the groups (experimental and control groups).

The types of VR devices used for the experimental groups in the studies varied considerably. Two studies used Wii training [29,32], and 1 study used the OpenFeasyo software platform (rehabilitation-specific gaming software) using the Kinect sensor and the Nintendo Wii Balance Board [30]. One study used the TYROMOTION force plate, which was specifically designed to assess and treat postural control [31], whereas another study used the RAPAEL Smart Kids [33], which was developed for rehabilitation purposes. Computer games with Lissajous feedback, targeting particularly bimanual coordination [35]; E-Link Evaluation and Exercise System [36]; and a computer game with a joystick [37] were used in the other studies. In one of the studies, the VR device was reported as a computer-assisted arm rehabilitation gaming technology [34]. Overall, most of the devices used in the studies (6/9, 67%) were specifically developed for therapy, whereas one-third (3/9, 33%) of the studies used commercially available VR devices. No safety issues or adverse events associated with VR training were reported.

The primary focus of VR interventions for most studies (7/9, 78%) was on the upper limb. Only 2 studies included trunk control training [30,31]. Similarly, the outcome measures in the studies included in this review were mainly related to upper limb functions and performance.

The duration of each VR session varied from 15 to 90 minutes, and the participants performed the VR intervention 2 to 7 times a week. In 1 study, participants’ compliance with the duration of VR interventions could not be fixed, and the mean daily duration of VR sessions was only 7 minutes [34]. As a result, the total amount of VR intervention of the included studies in this review was a minimum of 1 hour 39 minutes and a maximum of 20 hours.

In general (7/9, 78%), VR as the experimental group was mostly combined with conventional therapy and compared with a control group receiving only conventional therapy [30-36]. In one of these studies, there was >1 experimental group, with one group undergoing VR with conventional therapy (VR group) and the other following VR with modified constraint-induced movement therapy (mCIMT; VR + mCIMT group) [36]. One study applied VR therapy alone in the experimental group [29], whereas in another study, the experimental group participated in functional activities and the computer game while wearing the constraint (VR + mCIMT) [37].

The included studies had various outcome measures on motor functions and daily life activities at baseline (before intervention), after intervention, and at follow-up, which ranged from 4 to 12 weeks after completion of the interventions.

#### Upper Limb Functions

Overall, 78% (7/9) of the studies included in this review used a variety of outcome measures related to upper limb functions and performance. Furthermore, 3 studies used the Melbourne Assessment of Unilateral Upper Limb Function [29,33,37] and 2 studies used the ABILHAND-Kids questionnaire [29,34]. The Upper Limb Physician’s Rating Scale [33], Pediatrics Motor Activity Log, Speed and Dexterity subtest (subtest 8) of the Bruininks-Oseretsky test of Motor Proficiency [36], Nine-Hole Peg Test, Jebsen-Taylor Hand Function Test, Functional Use Survey [32], Assisting Hand Assessment [35], and Quality of Upper Extremity Skills Test [37] were also used in the studies.

Regarding the results of upper limb functions and performance, Kassee et al [29] did not conduct statistical analysis to determine group differences for the Melbourne Assessment and the ABILHAND-Kids questionnaire owing to the small sample size (n=3 for each group). Choi et al [33] and Psychouli and Kennedy [37] found a significant improvement in both experimental (VR with conventional therapy and VR with mCIMT, respectively) and control groups (conventional therapy and mCIMT, respectively) in the Melbourne Assessment after the intervention, but the VR groups showed more improvement in both studies. These improvements were retained at follow-up, after 4 or 8 weeks [33,37]. Moreover, Psychouli and Kennedy [37] found further improvement in the VR group in the Quality of Upper Extremity Skills Test after the intervention [37]. Rostamia et al [36] also demonstrated significant improvements in the experimental groups (VR and VR + mCIMT) in the Pediatrics Motor Activity Log and the Bruininks-Oseretsky test of Motor Proficiency but not in the control group (conventional therapy) [36]. The combined group (VR + mCIMT) showed the most significant improvements, which were maintained at the 3-month follow-up assessment [36].

Neither group improvements nor group differences in the ABILHAND-Kids questionnaire were found by Preston et al [34]. Chiu et al [32] did not find a difference between the experimental and the control group after the intervention or 6 weeks after the interventions in the Nine-Hole Peg Test and the Jebsen-Taylor Hand Function Test [32]. They also assessed elbow and finger coordination, and grip strength. As a result, there was a trend of mean difference for the experimental group to have more grip strength and Functional Use Survey (quantity) score [32]. In Assisting Hand Assessment scores at a group level, no significant improvements were observed by Peper et al [35].

#### Balance, Trunk Control, and Gross Motor Functions

Only 2 studies evaluated balance and trunk control using the Trunk Control Measurement Scale, Pediatric Balance Scale, or Pediatric Reach Test, and they also reported gross motor function results with Gross Motor Function Measure (GMFM; GMFM-66 or GMFM-88) [30,31]. Decavele et al [30] observed more improvement in Trunk Control Measurement Scale, Pediatric Balance Scale, and GMFM-88 after the intervention period (conventional therapy including rehabilitation-specific gaming) compared with the control period (conventional therapy), but the results did not persist after the 12-week follow-up [30]. Pin and Butler [31] found no significant difference in changes in the Pediatric Reach Test and the GMFM-66 between the 2 groups [31].

#### Daily Life Activities

Overall, 3 studies reported outcomes on daily life activities using the Goal Attainment Scale (GAS) [30], Pediatric Evaluation of Disability Inventory Computer Adaptive Test (PEDI-CAT) [33], or Canadian Occupational Performance Measure [34]. Decavele et al [30], in the GAS, and Choi et al [33], in the daily activities domain of the PEDI-CAT, found significant improvements in the VR group compared with the control group after intervention. The improvement in the PEDI-CAT was maintained at the 8-week follow-up [33] but not in the GAS at the 12-week follow-up [30]. Preston et al [34] found no significant differences in the changes on the Canadian Occupational Performance Measure between the 2 groups.

### Methodological Quality

All studies in this review (7 RCTs and 2 non-RCTs) were included in the methodological quality assessment. The total score of these studies ranged from fair to excellent. Of these 9 studies, 3 (33%) studies were classified as excellent-quality studies [32,34,36], 4 (44%) as good-quality studies [30,31,33,37], and 2 (22%) as fair-quality studies [29,35]. Table 4 presents the 4 main subscales and the total scores of the modified Downs and Black checklist. Scoring of all 27 items for the included studies is provided in Multimedia Appendix 2.

**Table 4:** Results of the quality assessment of included studies based on the modified Downs and Black checklist.

Study	Study type	Quality of reporting score (out of 11)	External validity score (out of 3)	Internal validity score (out of 13)	Statistical power score (out of 1)	Total score (out of 28)
Choi et al [33], 2021	RCT^a^	11	0	11	1	23
Preston et al [34], 2016	RCT	11	2	11	0	24
Peper et al [35], 2013	non-RCT	6	0	8	0	14
Rostami et al [36], 2012	RCT	10	2	12	0	24
Kassee et al [29], 2017	RCT	6	0	8	0	14
Psychouli and Kennedy [37], 2016	non-RCT	10	0	10	0	20
Decavele et al [30], 2020	RCT	10	0	10	1	21
Pin and Butler [31], 2019	RCT	9	2	11	0	22
Chiu et al [32], 2014	RCT	10	2	12	1	25

^a^RCT: randomized controlled trial.

### Results of Meta-Analysis

#### Overview

We performed meta-analyses with 6 RCTs supplying the essential data and classified them as good- to excellent-quality studies [30-34,36]. The upper limb function results of 4 studies were included in the meta-analysis [32-34,36], whereas results on balance, trunk control, and gross motor functions of only 2 studies were included [30,31]. Similarly, changes in daily life activities were documented in only 3 studies. However, as 1 study did not report the group mean scores separately [34], the results of 2 studies were used in the analysis [30,33]. In general, in all studies included in the meta-analysis, the results used for analyses were the postintervention and follow-up outcomes of the VR group (conventional therapy with VR training) and the control group (conventional therapy). Meta-analyses on follow-up results and subgroup analyses were conducted only for the studies with the outcomes of upper limb functions because there were enough studies (n=4) and results. We did not use any test to assess funnel plot asymmetry (reporting biases) because at least 10 studies included in the meta-analysis were required for meaningful results [38].

#### Effects of VR and Intervention of Control Group on the Upper Limb Functions of Children With CP From Short Term to Long Term

**Figure 2:**
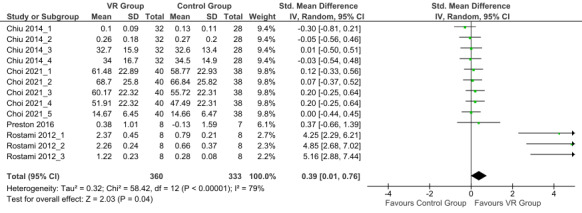
Forest plot of the postintervention upper limb functions outcomes [32-34,36]. Control group: conventional therapy; IV: inverse variance; VR group: virtual reality with conventional therapy.

The overall effect size was 0.39 (small effect; 95% CI 0.01-0.76; random effects model) with high heterogeneity (*I*^2^=79%). The results showed that after the intervention, overall effect on upper limb functions and performance in children with CP was in favor of the VR group when compared with the control group (*P*=.04; Figure 2 [32-34,36]).

**Figure 3:**
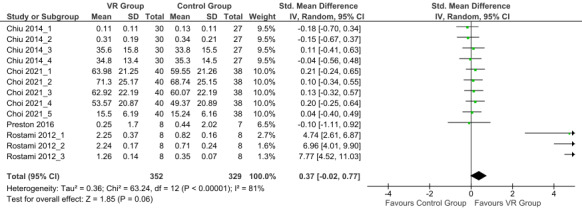
Forest plot of the follow-up upper limb functions outcomes (from 6 to 12 weeks after completion of the intervention) [32-34,36]. Control group: conventional therapy; IV: inverse variance; VR group: virtual reality with conventional therapy.

Concerning the medium- to long-term effects of interventions, the overall effect size was 0.37 (small effect; 95% CI −0.02 to 0.77; random effects model) with high heterogeneity (*I*^2^=81%). The overall effect showed a trend toward favoring the VR group 6 to 12 weeks after the completion of the interventions (*P*=.06; Figure 3 [32-34,36]), but the result was not statistically significant.

The subgroup analyses of both postintervention and follow-up results of upper limb functions showed that the duration and intensity of VR intervention and VR type were significant factors (*P*<.01). Studies that included more intensive and higher duration (≥60 min) of VR interventions had larger effects. Moreover, a larger effect was found when VR devices specifically developed for therapy were used compared with commercially available apparatuses. The figures are provided in Multimedia Appendix 3 [32-34,36].

#### Effects of VR and Intervention of Control Group on the Balance and Trunk Control of Children With BCP

**Figure 4:**
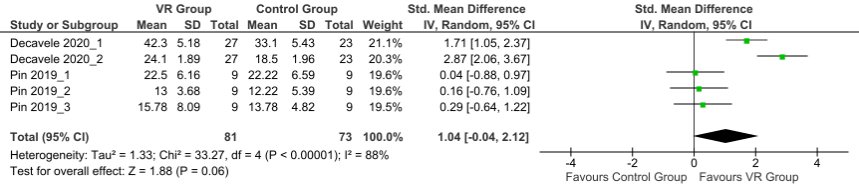
Forest plot of the postintervention balance and trunk control outcomes [30,31]. Control group: conventional therapy; IV: inverse variance; VR group: virtual reality with conventional therapy.

The overall effect size was 1.04 (large effect; 95% CI −0.04 to 2.12; random effects model) with high heterogeneity (*I*^2^=88%). The postintervention overall effect on balance and trunk control in children with BCP showed a trend toward favoring the VR group compared with the control group (*P*=.06; Figure 4 [30,31]), but the result was not statistically significant.

#### Effects of VR and Intervention of Control Group on the Gross Motor Functions of Children With BCP

**Figure 5:**
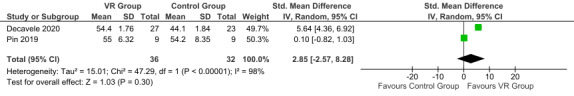
Forest plot of the postintervention gross motor functions outcomes [30,31]. Control group: conventional therapy; IV: inverse variance; VR group: virtual reality with conventional therapy.

The overall effect size was 2.85 (large effect; 95% CI −2.57 to 8.28; random effects model) with high heterogeneity (*I*^2^=98%). The postintervention overall effect on gross motor functions in children with BCP showed a trend toward favoring the VR group compared with the control group (*P*=.30; Figure 5 [30,31]), but the result was not statistically significant.

#### Effects of VR and Intervention of Control Group on the Activities of Daily Life of Children With CP

**Figure 6:**
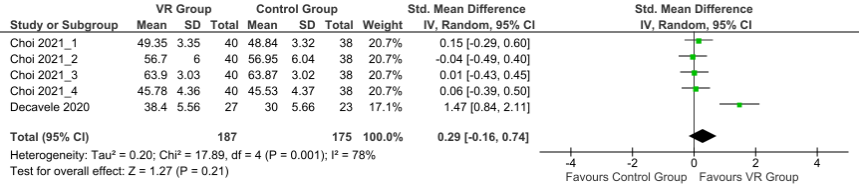
Forest plot of the postintervention activities of daily life outcomes [30,33]. Control group: conventional therapy; IV: inverse variance; VR group: virtual reality with conventional therapy.

The overall effect size was 0.29 (small effect; 95% CI −0.16 to 0.74; random effects model) with high heterogeneity (*I*^2^=78%). The postintervention overall effect on daily functions of children with CP showed a trend toward favoring the VR group compared with the control group. (*P*=.21; Figure 6 [30,33]), but the result was not statistically significant.

## Discussion

### Principal Findings

This systematic review and meta-analysis aimed to investigate whether VR-included therapies are effective on motor skill learning in children with CP compared with a control group, with special attention to the middle- and long-term effects. Overall, the results on upper limb function and performance showed the benefits of adding VR to CP rehabilitation in terms of postintervention improvements and supported that these acquisitions were mostly retained at follow-up. The meta-analysis results on upper limb functions and performance confirmed these benefits. In particular, Rostami et al [36] and Psychouli and Kennedy [37] used VR with mCIMT for 4 weeks, and greater improvements have been shown in the upper limb functions after VR with mCIMT compared with conventional therapy or mCIMT alone. Choi et al [33] matched VR with conventional occupational therapy for 4 weeks, and similarly, greater improvements have been shown in the upper limb functions following the intervention including VR compared with conventional occupational therapy alone [33]. In addition, these improvements were maintained at follow-up. It should also be noted that the contents of experimental interventions were shaped with motor skill learning principles (more intensity, the performance of daily living activities, repeating functional tasks, individually tailored, progressively challenging, and motivating feedback) in these 3 studies, which showed greater and long-lasting improvements in the VR groups (Table 2) [33,36,37]). Choi et al [33] and Rostami et al [36] applied these motor skill learning principles in VR game sessions, whereas Psychouli and Kennedy [37] used them mostly in mCIMT sessions and added a computer game as motivating feedback at the end of the intervention. Therefore, we concluded that incorporating motor skill learning principles into interventions including VR, through VR or therapy, induces greater benefits in motor function improvements in the long term. In addition, in the motor skill learning–based, VR-included interventions, VR has a substantially effect on maintaining motivation and eventually ensuring more intensity and repetition of child-initiated active movements during the interventions.

Furthermore, in line with our results from the subgroup analysis, applying more intensive VR training (involving more total hours in a shorter time, 4 weeks vs 6 weeks) had a significant influence on these larger improvements in the VR group both after the intervention and at follow-up compared with the other studies reporting no significant benefit of adding VR [32,34]. Thus, a higher intensity of VR training with either a longer duration or a higher frequency of training appears to be another essential factor for enhancing motor skill learning through VR. These findings were similar to the results of previous systematic reviews and meta-analyses [16,39-42]. They highlighted some principles of VR use in the neurorehabilitation of children with CP [13]. Rathinam et al [16] and Chen et al [39] also noticed in their reviews that more intensive VR training has more potential to result in improvements of functions in children with CP immediately after the intervention.

Furthermore, a study included in our review stated that parents were asked to encourage their children to use VR for 30 minutes daily. However, they played the games only for 7 minutes as a mean daily amount because of children’s low interest and motivation for the games in VR games [34]. This may explain why they did not find any improvement after VR-included therapy. In other words, maintaining interest and motivation also helps active participation in gaming in VR therapy and provides the expected therapy duration.

We also performed a subgroup analysis to determine whether VR types affected the upper limb function improvements. The results demonstrated that VR devices specifically developed for therapy have more potential benefits on the training of upper limb functions in children with CP when compared with the commercially available VR device (Nintendo Wii). In addition, VR devices specifically developed for therapy had a greater potential to make acquisitions persistent in the medium or long term. As explained by Chen et al [39], it seems more feasible to provide individually tailored interventions with active repetition in various contexts by integrating motor skill learning principles into VR devices when they are specifically developed for therapy purposes.

Concerning the outcomes of balance, trunk control, gross motor functions, and daily life functions induced by therapies combined with VR, we found only 2 studies that met the eligibility criteria of this systematic review and meta-analysis [30,31]. In addition, these 2 studies had different results. Decavele et al [30] found more improvement (though impermanent) after the VR combined period, whereas Pin and Butler [31] observed no significant difference between the groups. Even so, with the postintervention meta-analysis results of 2 good-quality studies, we showed that adding VR into therapy may offer additional benefits on balance and trunk control (SMD=1.04; *P*=.06) and likely in gross motor functions (SMD=2.85; *P*=.30) in children with BCP. Our findings were consistent with those of other reviews on balance and gross motor functions after VR interventions [18,43,44]. However, the very limited number of studies and small number of participants (<50) made it difficult to conclude the follow-up effects. Thus, it remains unclear whether there is more potential in VR-included therapy to facilitate long-lasting acquisitions in balance and gross motor functions in children with CP. The fact that both studies [30,31] only included children with BCP with GMFCS III-IV levels may also have affected the results. Further studies are needed to address the long-term effects of VR intervention on balance and gross motor functions in children with different severity of CP. The number of studies with long-term results that included daily functions was also very low (n=3) [30,33,34]. In fact, 2 out of 3 studies noted that changes in daily living activities after therapies with VR were significantly different between groups, with a tendency toward the VR group [30,33]. Similarly, the meta-analysis results were in favor of the VR group (SMD=0.29; *P*=.21). Considering that there are insufficient results on whether the acquisitions are long lasting, more studies on the daily function outcomes are also needed.

### Limitations

First, we found a limited number of studies and participants that met our inclusion criteria, especially for medium- and long-term outcomes. Only 7 RCTs and 2 non-RTCs were included in our systematic review, and most of them (7/9, 78%) had a relatively small sample size (<50). In addition, many studies did not report the MACS and GMFCS levels of the included children. The studies that reported the levels also included children with a relatively wide range of functional abilities. For instance, 1 study included children with GMFCS I-II level [29], whereas another study included children with GMFCS III-IV level [30]. As a result, this heterogeneity may have affected the results, but we could not perform a subgroup analysis because of the limited number of studies. Regarding the outcome measures, wide variations were used in the studies, and this heterogeneity of the outcome measures made it difficult to combine and interpret the results. Another limitation of this study is that if a study included in our review used different clinical tests to determine similar outcomes (upper limb functions, balance and trunk control, gross motor functions, and activities of daily living), all results were included in the analysis. Although the outcome measures were different, the intervention process of VR was identical in the same study. In addition, as one of the studies [30] had a crossover design, at the end of the study period, a control group (the group that did not receive VR) did not exist to allow comparison with the follow-up results of the VR group. Therefore, we were unable to conduct a meta-analysis of follow-up outcomes for balance, gross motor functions, and daily life activities, as there was only one study providing follow-up results on these outcomes.

### Conclusions

This review shows that adding VR into upper limb rehabilitation of children with CP offers additional benefits in motor skill learning and enhances the maintenance of obtained improvements when the VR included training that cooperates with motor skill learning principles, with the inclusion of functional tasks as well as repetitive practice, more intensity, individual adjustment, progressive challenge, and motivation. For these reasons, it appears more appropriate to make use of VR devices specifically developed for therapy. In terms of balance, trunk control, gross motor functions, and daily life activity outcomes, combining VR with therapies can help induce more improvements in children with CP. However, the priority focus of most of the interventions with VR (7/9, 78%) in the included studies, and their outcomes, was upper limb functions. The effect size on motor skill learning of adding balance-focused VR devices into different therapies in children with CP is still not entirely clear; therefore, studies with long-term outcomes are needed.

## Appendix

Multimedia Appendix 1 Search strategy.

Multimedia Appendix 2 Scoring of all 27 items of the modified Downs and Black checklist for the included studies.

Multimedia Appendix 3 Subgroup analyses.

Multimedia Appendix 4 PRISMA 2020 checklist.

Multimedia Appendix 5 PRISMA 2020 abstract checklist.

## Abbreviations

BCP: bilateral cerebral palsy
CP: cerebral palsy
GAS: Goal Attainment Scale
GMFCS: Gross Motor Function Classification System
GMFM: Gross Motor Function Measure
MACS: Manual Ability Classification System
mCIMT: modified constraint-induced movement therapy
PEDI-CAT: Pediatric Evaluation of Disability Inventory Computer Adaptive Test
PICO: Population, Intervention, Comparison, and Outcome
PRISMA: Preferred Reporting Items for Systematic Reviews and Meta-Analyses
RCT: randomized controlled trial
SMD: standardized mean difference
VR: virtual reality
