# Supplementary material for: Short- to Long-Term Effects of Virtual Reality on Motor Skill Learning in Children With Cerebral Palsy: Systematic Review and Meta-Analysis
Source: JMIR Serious Games. 2023 Sep 12;11:e42067. doi: 10.2196/42067 (PMC10523212; doi:10.2196/42067)
Supplement: Multimedia Appendix 1 [file games_v11i1e42067_app1.docx]

| **Databases** | **Combination of Keywords** |
| --- | --- |
| **Pubmed** | (virtual reality OR virtual environment OR videogaming OR computer game OR Kinect OR Wii OR Playstation) AND (cerebral palsy OR CP) AND (rehabilitation OR therapy OR motor skill learning) |
| **Science Direct** | ("virtual reality" OR "virtual environment" OR "videogaming" OR "computer game" OR "Kinect") AND ("cerebral palsy" OR "CP") AND ("rehabilitation" OR "therapy") |
| **Embase** | ("virtual reality" OR "virtual environment" OR "videogaming" OR "computer game" OR "Kinect" OR "Wii" OR "Playstation") AND ("cerebral palsy" OR "CP") AND ("rehabilitation" OR "therapy" OR "motor skill learning") |
| **IEEE Xplore** | ("virtual reality" OR "virtual environment" OR "videogaming" OR "computer game" OR "Kinect" OR "Wii" OR "Playstation") AND ("cerebral palsy" OR "CP") AND ("rehabilitation" OR "therapy" OR "motor skill learning") |

**Appendix 1.** Search strategy
