# Supplementary material for: Short- to Long-Term Effects of Virtual Reality on Motor Skill Learning in Children With Cerebral Palsy: Systematic Review and Meta-Analysis
Source: JMIR Serious Games. 2023 Sep 12;11:e42067. doi: 10.2196/42067 (PMC10523212; doi:10.2196/42067)

**Appendix 3.1.** Subgroup analyses of post-intervention results according to the duration of VR
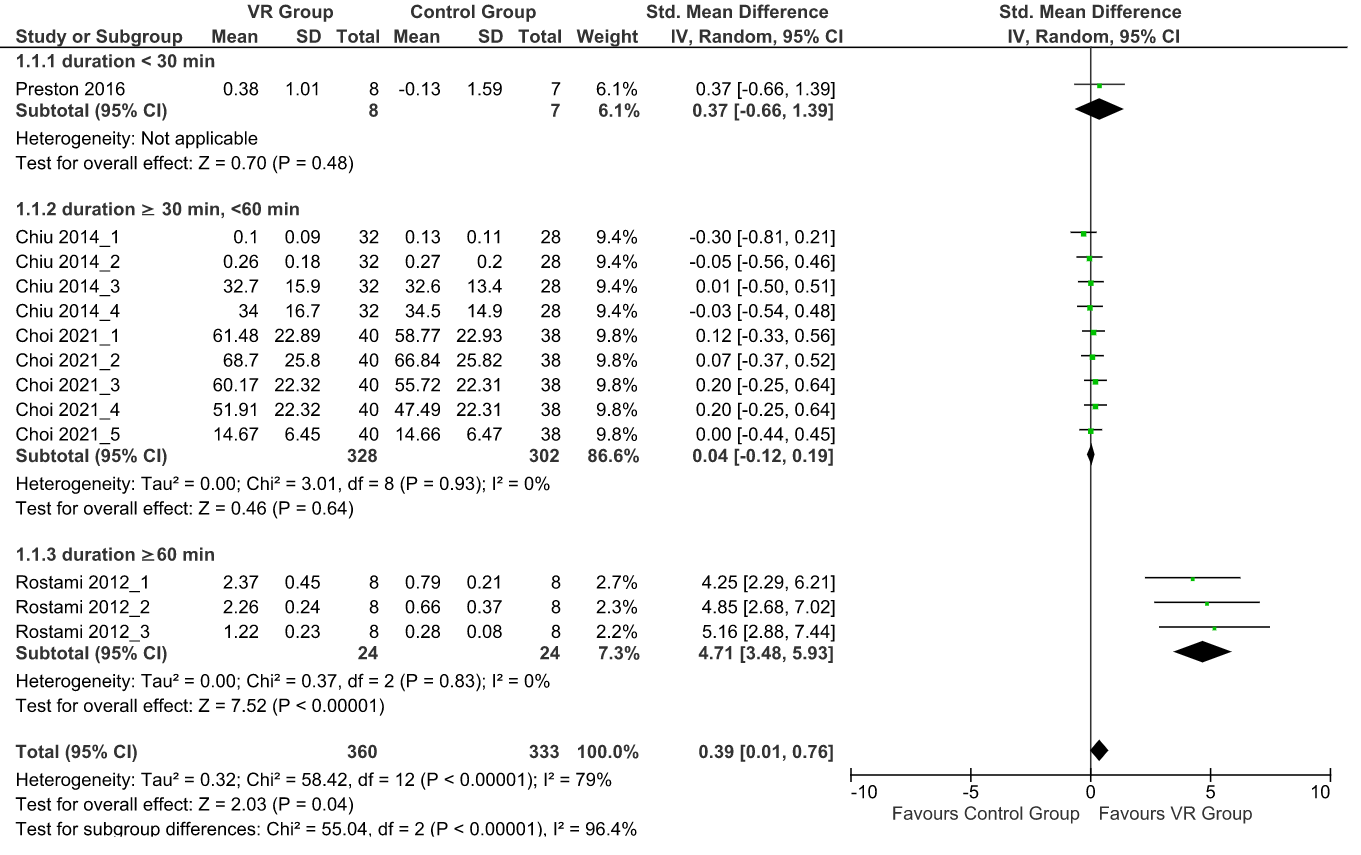


**Appendix 3.2.** Subgroup analyses of post-intervention results according to the intensity of VR


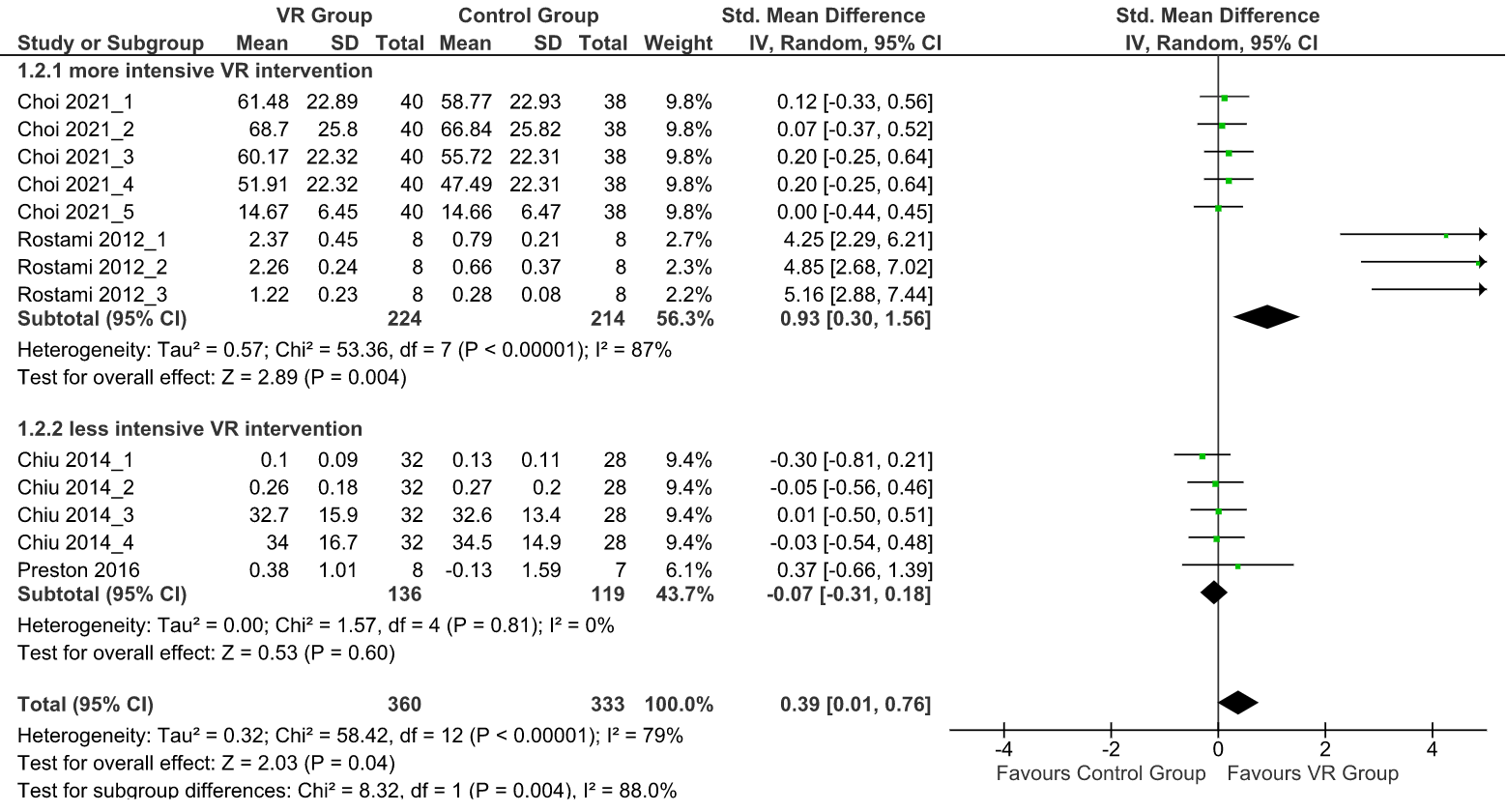


**Appendix 3.3.** Subgroup analyses of post-intervention results according to the VR type


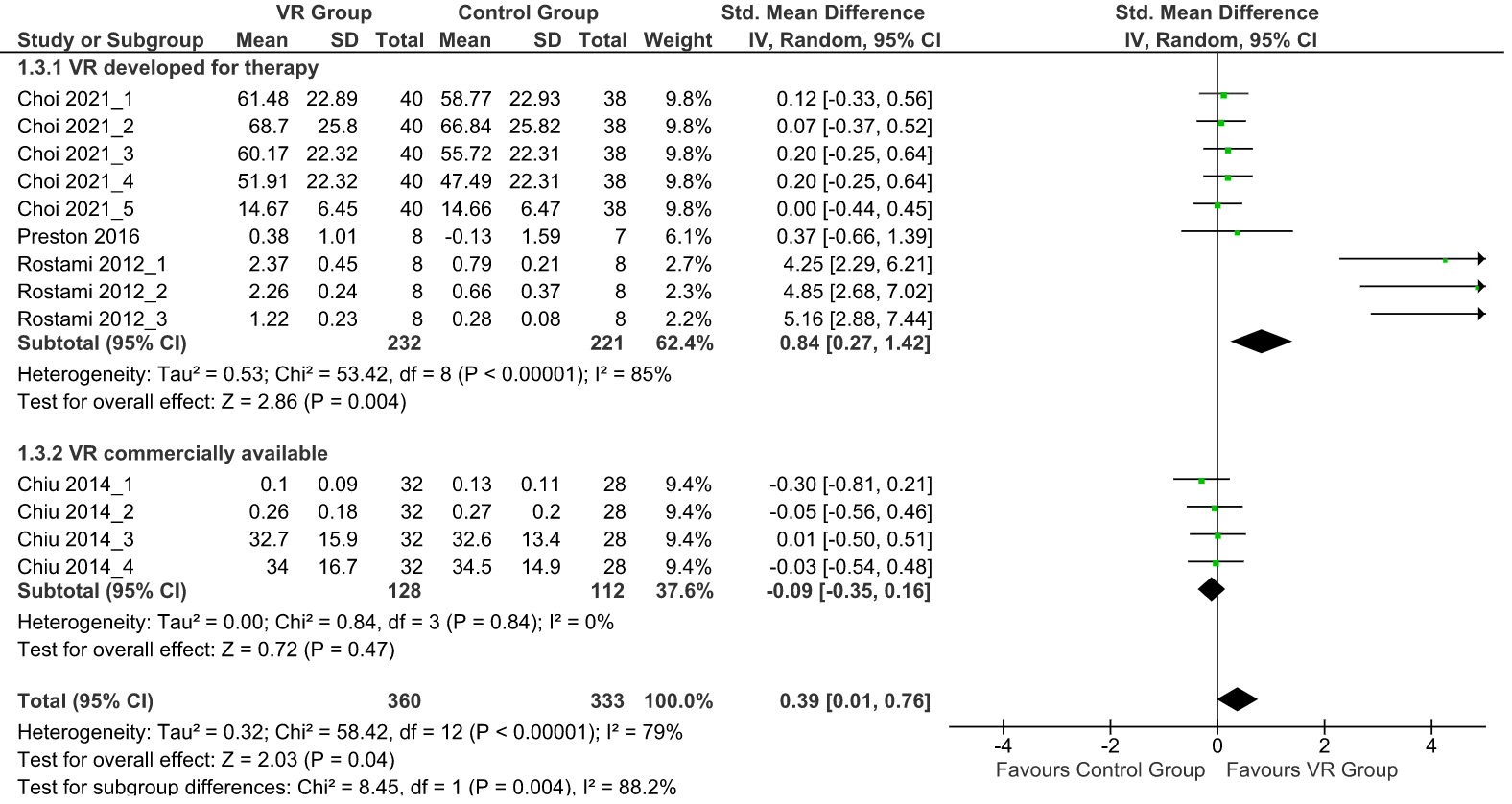


**Appendix 3.4.** Subgroup analyses of follow-up results according to the duration of VR


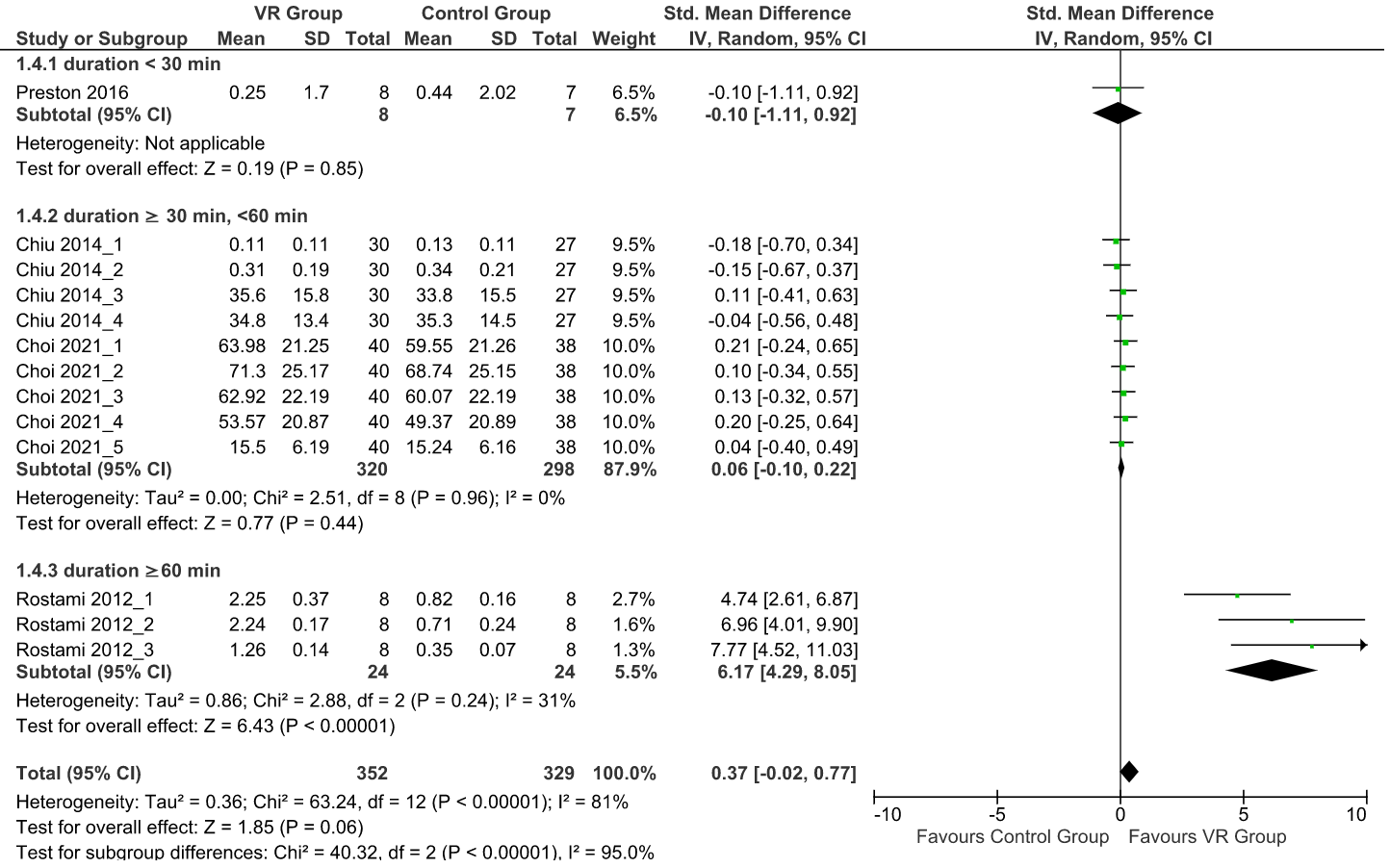


**Appendix** **3.5.** Subgroup analyses of follow-up results according to the intensity of VR


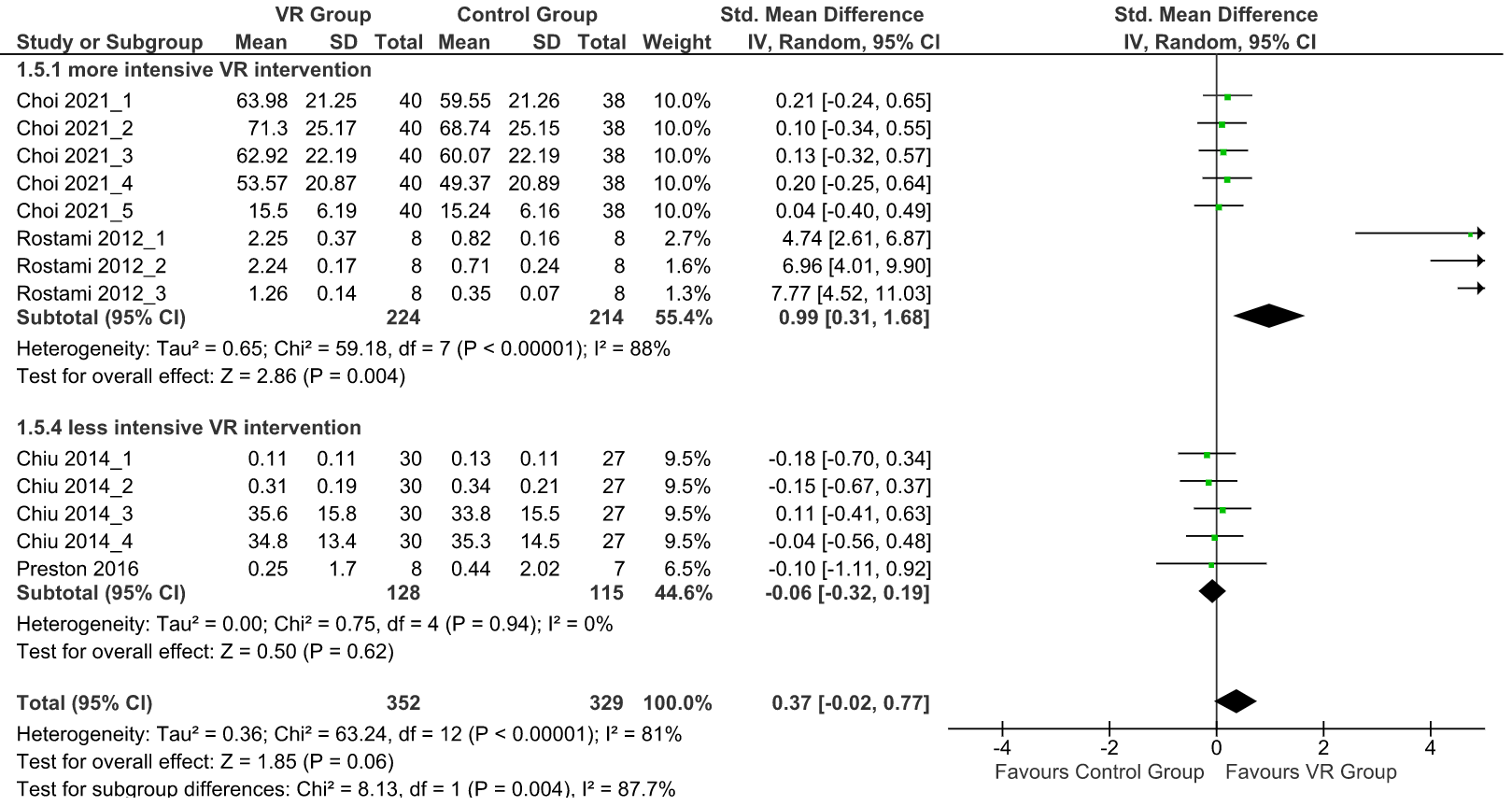


**Appendix** **3.6.** Subgroup analyses of follow-up results according to the VR type


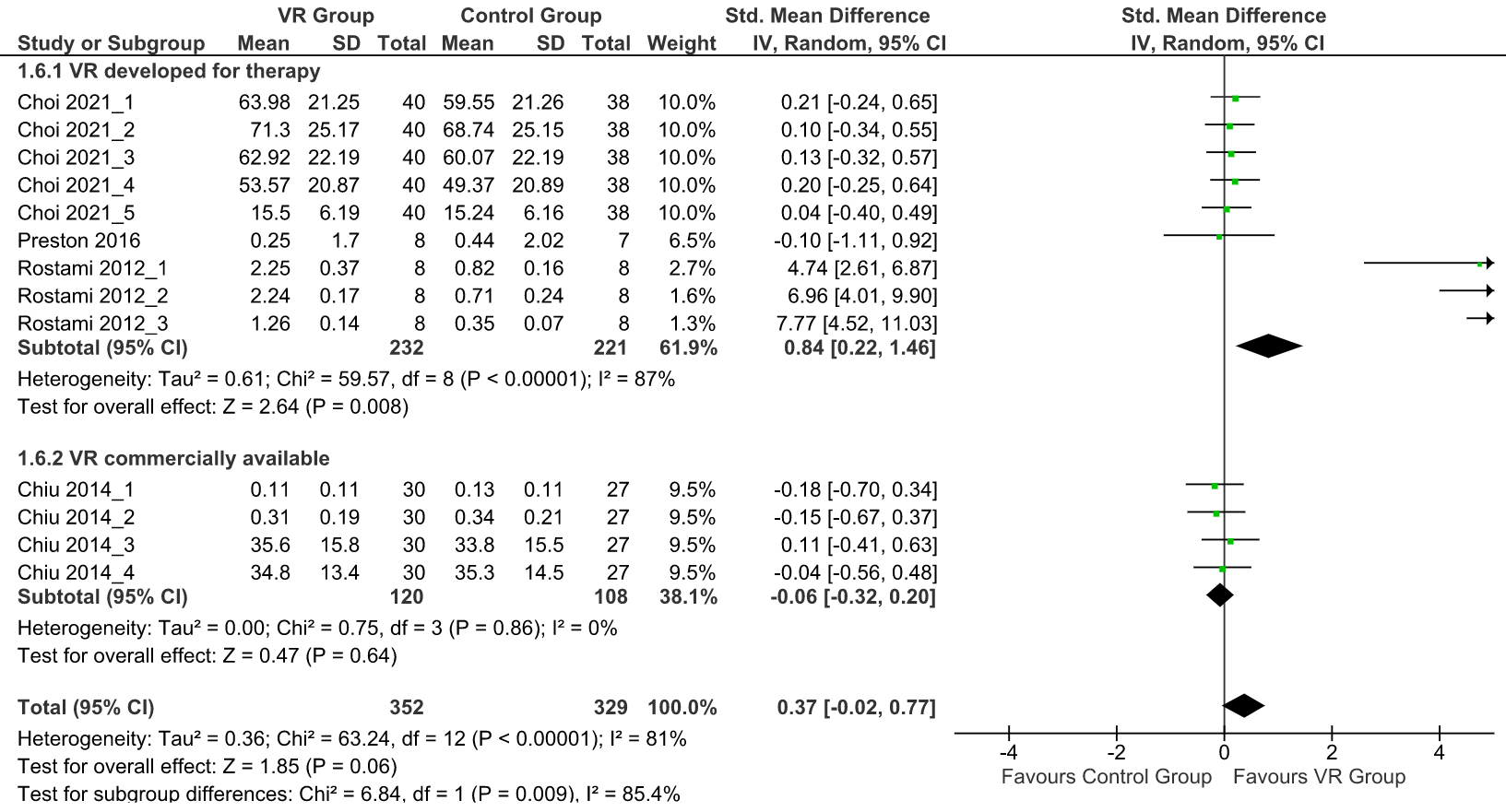

Supplement: Multimedia Appendix 3 [file games_v11i1e42067_app3.docx]
